# Equifinal pathways and subtypes: a developmental neuroimaging perspective on social differences in autism

**DOI:** 10.3389/fpsyt.2026.1919681

**Published:** 2026-09-28

**Authors:** Yuanqi Cai, Lara Maliske, Philipp Kanske

**Affiliations:** 1 Clinical Psychology and Behavioral Neuroscience, Institute of Clinical Psychology and Psychotherapy, Faculty of Psychology, TUD Dresden University of Technology, Dresden, Germany; 2 Department of Psychology, Faculty of Psychology and Educational Sciences, Babeş-Bolyai University, Cluj-Napoca, Romania

**Keywords:** autism spectrum disorder, fMRI, neuroimaging, social cognition, social difference

## Introduction

Social differences are a defining feature of autism spectrum disorder (ASD) (1). Autistic individuals show differences across multiple domains of social processing, including reciprocal behavior, social communication, and interpretation of facial expressions (2, 3). While social behavior in autistic individuals differs from that of non-autistic peers, some of these differences may also be context-dependent and reflect adaptive strategies given underlying sensory and cognitive profiles (4). Various studies have probed the neural mechanisms underlying these differences. Self- and caregiver-report studies suggest differences in social motivation in autistic individuals (5, 6), and neuroimaging evidence implicates reward-related regions such as the ACC, vmPFC, OFC, amygdala, insula, and striatum (7). An alternative view emphasizes differences in understanding others’ perspectives, associated with the mentalizing/Theory of Mind processes (8), including medial prefrontal cortex (mPFC), temporoparietal junction, and dorsomedial prefrontal cortex (9, 10). Additionally, co-existing hyper- and hypoconnectivity in social brain regions (11) point to the roles of developmental timing and the specificity of certain brain regions in influencing connectivity patterns in ASD (12). This lack of generalizability reveals the biological and behavioral complexity of ASD (13).

To reconcile these co-existing hypotheses, our opinion article introduces a two-dimensional framework—comprising functional operations and developmental spatiotemporal specificity—by selectively synthesizing evidence primarily from MRI studies published over the past decade. We suggest that these mixed empirical findings may stem from the intrinsic heterogeneity of ASD, which gives rise to multiple potential developmental pathways. Consequently, ostensibly similar social differences in ASD may represent a common behavioral outcome arising from distinct developmental alterations that progressively shape specialized brain networks.

## Neuroimaging in ASD: two dimensions of evidence

Within our two-dimensional framework (**Figure 1**), the *first dimension* focuses on functional operations, investigating whether autistic individuals exhibit atypical neural responses for distinct components of social processing and other basic processes that shape social behavior. The social motivation hypothesis posits that autistic individuals show atypical patterns during social reward processing. A meta-analysis by Clements et al. (7), based primarily on autistic males from childhood to adulthood without intellectual disability, reported reduced striatal responses to social and non-social rewards. However, a systematic review across multiple measures found the evidence for this hypothesis to be mixed, as reward-related differences vary by reward type, task, and brain region (14).

**Figure 1:**
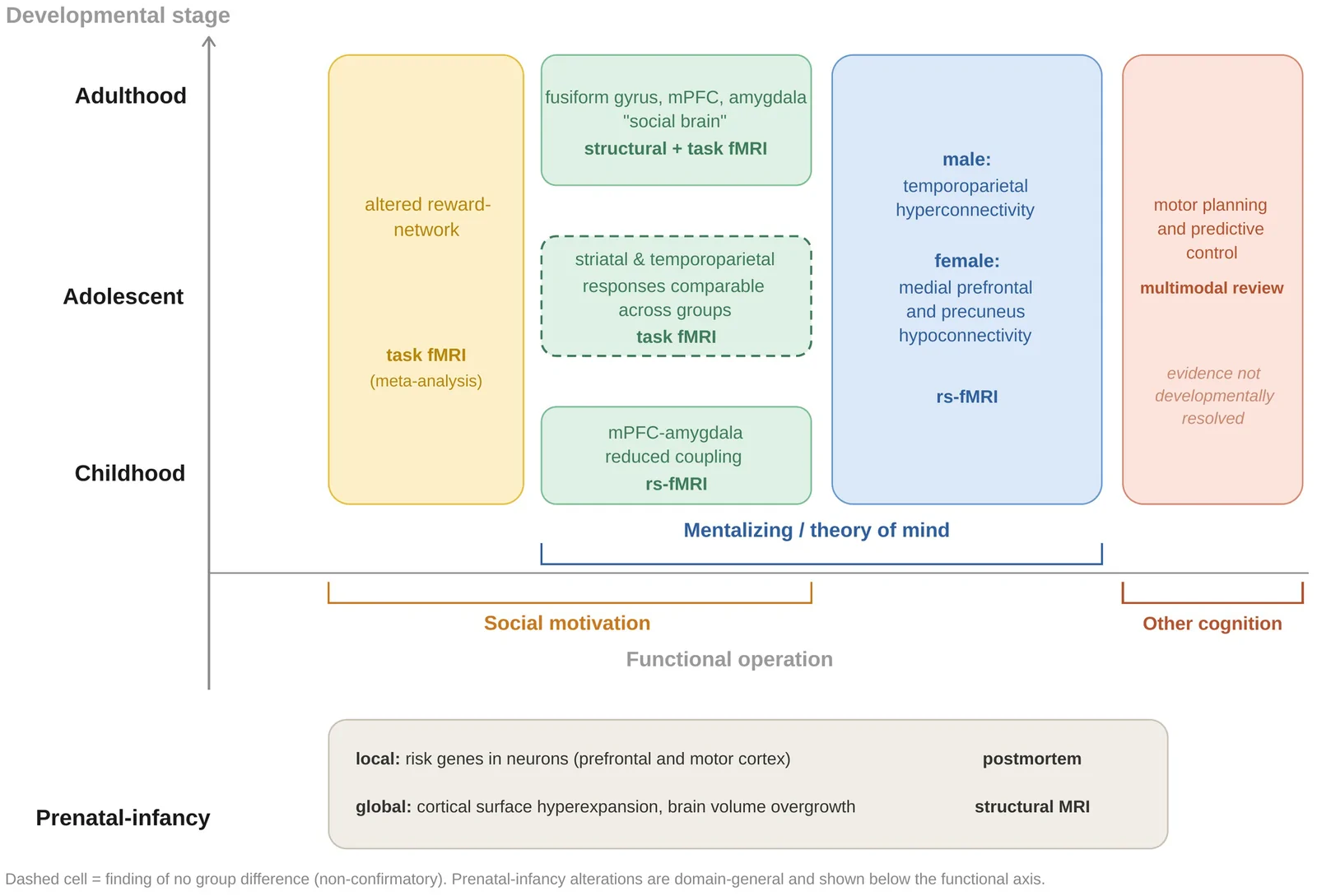
The two-dimensional framework. The vertical axis represents developmental stage; the horizontal axis represents functional operations. Three operations are distinguished by color: social motivation (yellow), mentalizing/theory of mind (blue), and motor prediction and other cognitive functions (coral); green marks regions where the social motivation and mentalizing/theory of mind operations overlap. Solid outlines denote supported findings; the dashed cell denotes a non-confirmatory finding (no group difference). Prenatal-infancy alterations are not functionally specific and are therefore placed below the functional axis rather than assigned to a single operation. fMRI, functional magnetic resonance imaging; mPFC, medial prefrontal cortex; MRI, magnetic resonance imaging; rs-fMRI, resting-state functional MRI.

Alternatively, the mentalizing differences hypothesis proposes that social differences primarily stem from alterations in mentalizing abilities, supported by task-based evidence including only male adults (15). However, a large cross-sectional sample spanning childhood to adulthood reveals that IQ-matched autistic males and females exhibit distinct mentalizing-network resting-state connectivity, with temporoparietal hyperconnectivity in males but medial prefrontal and precuneus hypoconnectivity in females (16).

Beyond these specifically social processes, a review integrating behavioral, electrophysiological, and neuroimaging evidence argues that atypicalities in other cognitive functions, such as motor planning and predictive control can also contribute to social differences in ASD (17). These foundational alterations may lead to differences in action understanding and social initiation (17–19). Together, these findings show how atypicalities in partially distinct functional operations—reward, mentalizing, motor prediction as illustrative examples—can each manifest as social differences.

The *second dimension* maps neurodevelopmental trajectories, examining how social phenotypes in ASD link to specific developmental windows and brain regions. Prenatally, certain types of ASD risk genes were found converging in deep-layer projection neurons of the prefrontal and motor cortices across both sexes via post-mortem analysis (20), suggesting early vulnerability of local regions, though this does not rule out alterations in other brain regions. Structural studies of high-familial-risk infant cohorts show that, during the first year, infants who later receive an ASD diagnosis exhibit hyperexpansion of cortical surface area (21). These systemic alterations in perceptual, attentional, motor, and social domains often emerge before overt ASD behavioral traits manifest (22). In the second year and beyond, overall brain volume overgrowth was detected, correlating with the severity of later social differences (21, 23).

Together, current findings suggest that neurodevelopmental alterations unfold across multiple developmental stages. This developmental perspective also provides a time window for interpreting the functional alterations described in the first dimension. For autistic children, resting-state evidence shows attenuated effective connectivity between the mPFC and amygdala, which are nodes shared by the mentalizing and reward networks (24). By early adolescence, task-based interactive social reward elicited comparable striatal and temporoparietal responses across autistic and neurotypical groups (25), so neither reward- nor mentalizing-related regions showed differences in this paradigm. In autistic adults, social differences may also involve multimodal structural and functional atypicalities within the fusiform gyrus, mPFC, and amygdala (26). These regions are components of the ‘social brain’, which support processes including reward anticipation and mentalizing (27). Beyond these specialized circuits, multimodal evidence links autistic social differences to alterations across widespread networks (28) and reduced global efficiency in the white matter connectome (29).

Across these developmental stages, we propose one possible pattern—both global and localized brain alterations may coexist in early life, with atypicalities gradually emerging in higher-level functional networks as social function develops with age. However, the dominant imaging modality differs across age, so developmental shifts remain partly confounded with method. Cross-study comparisons are further limited by methodological variability—task design, head motion, medication, and scanner effects—and by the largely cross-sectional design, with longitudinal data in infancy coming from high-familial-risk cohorts whose findings may not extend to the broader autistic population. Participant samples also rarely include individuals with intellectual disability, and sex shapes mentalizing-network alterations (16). Such variations, however, are unlikely to fully explain the inconsistencies. The remainder points to genuine within-diagnosis heterogeneity.

## From heterogeneity to equifinality: rethinking social differences in ASD

Our framework illustrates how heterogeneity—the biological diversity within ASD—leads to equifinality, where different developmental pathways converge onto a similar, clinically defined social phenotype. One possible explanation for the inconsistencies is the etiological heterogeneity within autistic populations (28, 30). For instance, Rødgaard et al. (31) observed a longitudinal decrease in differences between autistic and neurotypical participants from 1966 to 2019. This narrowing partly indicates broadening diagnostic criteria and highlights heterogeneity within ASD.Our framework aligns with the evidence that diverse etiological mechanisms can lead to generalized ASD traits (32, 33). This equifinality explains why heterogeneity is a barrier to understanding social differences in ASD, as a single clinical diagnosis often masks highly diverse biological pathways.

Several hypotheses investigate why ASD shows this equifinality in social differences. Johnson (34) proposed that some autistic behaviors may reflect early-life adaptation to atypical neural signal processing. For example, social withdrawal may serve as an adaptive strategy to limit sensory and cognitive input when the brain shows atypicalities in handling multidimensional, parallel, and dynamic social stimuli. On this account, the social brain network’s high metabolic demand and reliance on long-range integration may render it particularly vulnerable to subtle synaptic alterations. Because such adaptation depends on compensation, environment, and learning, similar early alterations need not lead to the same outcome; the framework thus describes probabilistic rather than fixed developmental routes.

Additionally, the cascade hypothesis suggests that the foundational alterations may result in missed critical social learning opportunities. For example, infants’ reduced object manipulation may limit the caregiver’s language input, while atypical sensory attention can affect social cue recognition. Consequently, these initial disruptions may lead to social differences (35, 36). If diverse pathways converge on similar social outcomes, the research task becomes to delineate these distinct pathways—that is, to identify meaningful biological subtypes. Our framework makes a further prediction: autistic individuals matched on similar social-communication profiles are predicted to show distinguishable longitudinal network trajectories.

Classifying more homogeneous subgroups within ASD may address this heterogeneity (31, 37). For example, Litman et al. (38) identified four genetic-developmental subtypes. These subtypes—moderate challenges, broadly affected, social or behavioral, and mixed ASD with developmental delay—correlated with specific sets of genetic alterations acting at variable developmental windows (prenatal to adolescence). In terms of brain networks, Lombardo et al. (39) identified a subtype that features early visual-social engagement differences, associated with hypoconnectivity between the default mode network and occipito-temporal cortex. At the micro-level, subtype-specific gene sets are linked to different molecular signaling pathways, involving immune and synapse function, G-protein-coupled receptor signaling, and protein synthesis (40). Taken together, while these criteria of classification vary, they may not be mutually exclusive. In fact, this evidence depicts several potential links on how developmental timing, as well as genetic or neural pathways shape ASD phenotypes.

## Discussion

Our framework locates social differences in ASD within interacting networks that develop over time, rather than in fixed core regions. We propose that social differences should be viewed as the convergent outcome of various alterations across a wide range of neural networks. Importantly, the so-called social brain is increasingly viewed as an interconnected ‘social connectome’ including cooperating and overlapping networks (41–43). Because social cognition draws on brain regions involved in perception, motor skills, and attention (44), alterations in these foundational levels can cascade upward to social differences (36). Unlike models that describe how these alterations arise or spread, our framework maps where to look for them—at which developmental stage and in which operation.

Future work should consider developmental timing to explore whether these atypical developments are due to prenatal or genetic factors, or to the cascading effects of synaptic pruning. In addition, rather than focusing solely on one mechanism that fits the broad ASD criteria defined by behavioral traits, future studies should emphasize identifying subtypes characterized by divergent neural developmental trajectories. Indeed, recent advances have identified distinct ASD subtypes based on brain connectivity patterns and developmental timing (38, 45, 46).

However, valid ASD subtypes should reflect coherent neurodevelopmental pathways rather than mere statistical clusters, which are sensitive to analytical choices. More fundamentally, whether heterogeneity is discrete or continuous remains an open question for future research. Ideally, these pathways should exhibit longitudinal stability and neurobiological coherence across developmental trajectories and show external clinical validity, including the ability to predict behavioral profiles and intervention response. Understanding the neurodevelopmental evolution of these subtypes could then enhance the interpretability and replicability of studies and guide precise interventions (45, 47).

Such subtyping efforts may facilitate more tailored interventions for specific autistic subgroups. For instance, preliminary evidence suggests that some autistic individuals with reward-network alterations may benefit from interventions targeting social reward processing (48), although whether reward-network-defined subtypes predict treatment response remains to be established. Developmental timing is also critical, as evidence supports the importance of early intervention during periods of heightened neuroplasticity (35, 36, 49), despite limited evidence for imaging-defined subtype-specific early interventions.

Finally, because social differences can emerge as an adaptive response to reduce cognitive load (34), interventions should be carefully evaluated for potential anxiety, distress, or other unintended effects, given concerns about incomplete adverse-event reporting (50). Therefore, interventions should support sensory and cognitive mechanisms while also considering an autistic individual’s support level. For now, our framework guides future stratification research rather than clinical decision-making.

To sum up, we argue that embracing equifinality and mapping functional operations onto developmental spatiotemporal trajectories can enable biologically grounded subtyping and, ultimately, more humane, individualized support that prioritizes autonomy and quality of life over the normalization of social behavior.
